# Role of Respiratory Epithelial Cells in Allergic Diseases

**DOI:** 10.3390/cells11091387

**Published:** 2022-04-20

**Authors:** Constanze A. Jakwerth, Jose Ordovas-Montanes, Simon Blank, Carsten B. Schmidt-Weber, Ulrich M. Zissler

**Affiliations:** 1 Center of Allergy and Environment (ZAUM), Technical University of Munich and Helmholtz Center Munich, German Research Center for Environmental Health, Germany, Member of the German Center for Lung Research (DZL), Member of the Immunology and Inflammation Initiative of the Helmholtz Association, 80802 Munich, Germany; constanze.jakwerth@tum.de (C.A.J.); simon.blank@tum.de (S.B.); csweber@tum.de (C.B.S.-W.); 2 Division of Gastroenterology, Hepatology, and Nutrition, Boston Children’s Hospital, Boston, MA 02115, USA; jose.ordovas-montanes@childrens.harvard.edu; 3 Program in Immunology, Harvard Medical School, Boston, MA 02115, USA; 4 Broad Institute of MIT and Harvard, Cambridge, MA 02142, USA; 5 Harvard Stem Cell Institute, Cambridge, MA 02138, USA; 6 Ragon Institute of MGH, MIT and Harvard, Cambridge, MA 02139, USA

**Keywords:** epithelial immunity, asthma, allergy, airways, airway lining fluids

## Abstract

The airway epithelium provides the first line of defense to the surrounding environment. However, dysfunctions of this physical barrier are frequently observed in allergic diseases, which are tightly connected with pro- or anti-inflammatory processes. When the epithelial cells are confronted with allergens or pathogens, specific response mechanisms are set in motion, which in homeostasis, lead to the elimination of the invaders and leave permanent traces on the respiratory epithelium. However, allergens can also cause damage in the sensitized organism, which can be ascribed to the excessive immune reactions. The tight interaction of epithelial cells of the upper and lower airways with local and systemic immune cells can leave an imprint that may mirror the pathophysiology. The interaction with effector T cells, along with the macrophages, play an important role in this response, as reflected in the gene expression profiles (transcriptomes) of the epithelial cells, as well as in the secretory pattern (secretomes). Further, the storage of information from past exposures as memories within discrete cell types may allow a tissue to inform and fundamentally alter its future responses. Recently, several lines of evidence have highlighted the contributions from myeloid cells, lymphoid cells, stromal cells, mast cells, and epithelial cells to the emerging concepts of inflammatory memory and trained immunity.

## 1. Introduction

The nature of the epithelium changes in different regions of the airways. In the upper airways, a pseudostratified columnar epithelium is dominating [1]. In the lower airways, namely the trachea and bronchi, the pseudostratified columnar epithelium is transitioned into cuboidal cells in the bronchioles, forming a single-layer alveolar epithelium [2,3]. The alveolar epithelium, which is responsible for gas exchange, receives air from conducting airways. The alveolar epithelium consists of two subtypes of alveolar cells. Type 1 (AT1) epithelial cells are flat-shaped epithelial cells, which are responsible for the transfer of oxygen into the periphery, while type 2 (AT2) epithelial cells are cuboidal-shaped and progenitor cells for AT1 cells [4]. They produce surfactants to reduce the surface tension at the air-liquid interface of the lung, playing a pivotal role as mediators of various immune-cell functions involved in anti-viral mechanisms [5]. The pseudostratified epithelial layer of the conducting airways is separated from the underlying mesenchyme by the basal membrane. It consists of basal epithelial cells, club cells, goblet cells, ciliated cells, and rare cells such as ionocytes and pulmonary neuroendocrine cells (PNECs) [6]. Airway basal epithelial cells are progenitors with the ability to differentiate into secretory club cells. These can further differentiate into mucus-producing goblet cells or mucus-clearing ciliated cells [7]. Club cells are capable of self-renewing and generating ciliated cells to repopulate damaged airway tissue. Secretory cells can dedifferentiate into basal cells, underscoring the remarkable plasticity of the airway epithelium [8]. While some studies have shown that ciliated cells are terminally differentiated [9], others have demonstrated that ciliated cells can undergo dynamic changes in cell shape and gene expression to re-differentiate into columnar cells upon naphthalene-induced injury [10]. In the presence of IL-4 or IL-13, ciliated cells can transdifferentiate into goblet cells [11]. Small, non-coding microRNAs (miRs) are also affected by these mechanisms involved in airway immunology and homeostasis.

In allergic airway diseases, allergens cross-link specific immunoglobulin E (IgE) bound to receptors on the surface of immune effector cells, which leads to the characteristic symptoms in allergic individuals [12]. Allergic asthma is a very common form of allergic respiratory disease, with atopy and allergic sensitization identified as the main risk factors [13]. However, in addition to allergic triggers, risk factors such as viral infections, family history, environmental exposure, smoking, and obesity also play an important role in the development of allergic asthma in children [14,15]. In addition to increased levels of IgE, the features of allergic diseases also include the chronicity of the inflammatory reaction, which is caused by the type 2 key cytokines inducing changes in the structural components of the airway wall, such as airway smooth muscle cells and fibroblasts. There is also goblet cell hyperplasia and, as a direct consequence, mucus hypersecretion in allergic asthma.

## 2. Immune Cell Interactions with the Epithelial Surface

### 2.1. Overview

**Figure 1:**
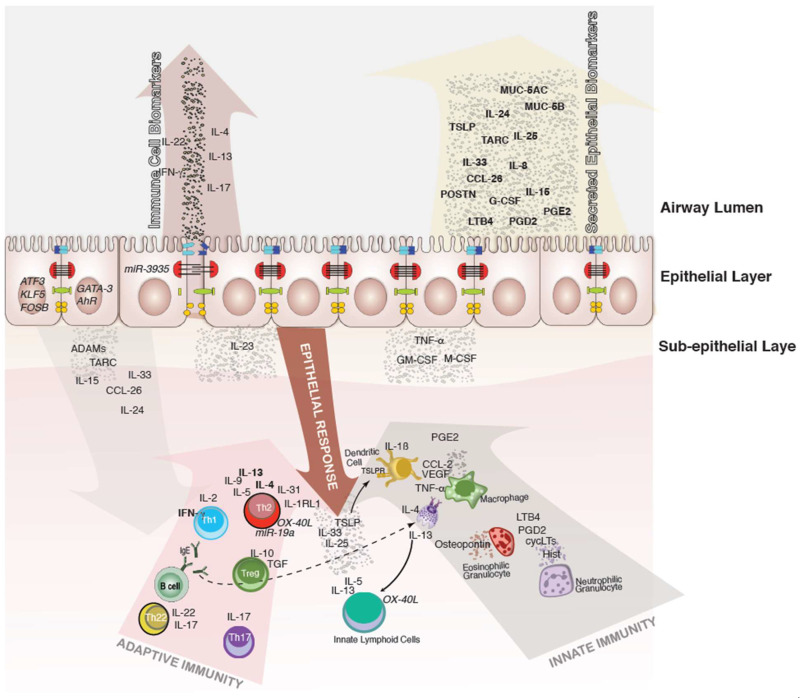
Schematic overview on biomarkers involved in immunological processes of the epithelial airway barrier. The airway lumen unifies cytokines and mediators of different origins, from immune cells as well as the airway epithelium. While cytokines produced by immune cells transmigrate through the epithelial barrier and can be detected indirectly, mediators produced and secreted by airway epithelial cells have a more direct access. However, epithelium-derived mediators can be secreted on the apical surface, as well on the basal layer. Airway epithelial cells are pre-committed to a type 2 (E2) or type 1 (E1) like phenotype, resulting from a priming effect of airway epithelial cells, which was named after the causative Th1 versus Th2-derived cytokines. E2 epithelial cell activation by allergens takes place and their pro-inflammatory cytokines and chemokines induce inflammation, contributing to an epithelial type 2 response, the so called “E2 response,” with epithelial alarmins TSLP, IL-31, CCL-26, IL-25, and IL-33. Immune cell-derived type 2 responses involve multiple cytokines, including IL-4, IL-5, IL-9, IL-13, IL-33, and increased eosinophil numbers. Bronchial hyperreactivity takes place, leading to an enhanced susceptibility to bronchoconstriction mediated by, e.g., IL-13. E1 epithelial cells arise as a response to, e.g., viral infections secreting CXCL2, CXCL8, IL12, CCL2, and CCL20, thus stimulating the local synthesis of IFN-γ, IL-2, IL-12, IL-18, IL-36, and TNF-a that present a wide range of antiviral activities, inducing the upregulation of MHC-I molecules and antiviral resistance in uninfected cells. Neutrophils respond to the infection signals IL-12 and IFN-γ by releasing pro-inflammatory cytokines, which leads to the limitation of the infection, rise of body temperature, and to the recruitment of further phagocytic cells. Upon allergen exposure, airway epithelial cells are activated by a variety of receptors, releasing chemoattractant mediators and recruiting a variety of immune cells to the airways, leading to an increased mast cell-derived release of IL-13 and TNF, which play a crucial role in inducing the mucus production by goblet cells.

Airway epithelial cells act in an orchestrated manner with resident and recruited immune cells to regulate airway immunity (Figure 1). Whilst transcriptomic studies of the local matrices of the airways, such as the extracellular matrix, but also induced sputum [16,17,18] or bronchoalveolar lavage (BAL) [19,20], facilitate the detailed characterization of immune cell populations within the airway lumen, bronchial brushing samples and biopsies of the upper and lower airways from allergic patients and healthy subjects—even if ethically justifiable only with indication—capture both the epithelial and immune cell components. The concept of united airways encompasses an anatomical and immunological relationship between the upper and lower airways, which form a single organ. The concept combines diseases of the upper and lower respiratory tract, which according to this concept, often have comorbidities, since they reflect manifestations of a single underlying disease at different points in the respiratory tract. Allergic asthma as a comorbidity of allergic rhinitis represents the archetypal “united airways” disease, but could also be a heterogeneous condition consisting of several phenotypes and underlying pathobiological mechanisms [16].

The latest technical advances, such as single-cell RNA-sequencing (scRNA-seq), can currently be used to unravel the complex epithelial–immune crosstalk that occurs in the airways and alveolar space in both health and disease [21,22,23].

### 2.2. T Cells

The allergic immune response of the respiratory epithelium to environmental factors such as pollen, house dust mites, and animal dander is driven primarily by means of an adaptive T-cell response. In allergic patients, the balance between the T helper cell response of type 1 (Th1) and type 2 (Th2) is shifted in favor of the Th2 cells [24,25,26]. From these type 2 mediators, IL-4 triggers class switch recombination in mature B cells from IgM to IgE, which also requires co-stimulatory signals and activation by means of CD40/CD40L [27,28]. IL-5 and IL-13 trigger the recruitment of eosinophilic granulocytes and mast cells into the mucosa of the airways. Remodeling processes of the airways [29], including phenomena such as goblet cell hyperplasia [30], increase the risk of local hypersensitivity reactions [31]. Interestingly, regulatory molecules, the inhibitory receptors PD-1 and CTLA-4, are upregulated on the Th2 cells in the sputum of symptomatic allergic patients during the pollen season, compared to healthy controls, due to chronic T cell receptor stimulation that drives the Th2 cells into exhaustion [32]. However, further studies identified oscillating levels of regulatory T cells (Tregs), as well as effector T cells dependent on allergen exposition in and out of pollen season, highlighting the importance of Treg antigen-specificity for tolerance in humans and identifying antigen-specific escape from Treg control as an important mechanism enabling antigen-specific loss of tolerance in human allergy [33].

### 2.3. Th17 Cells

In addition to the shift to a type 2 immune response, Th17 cells, characterized by their secretion of IL-17A (IL-17), also contribute locally in the mucosa to the allergic airway inflammation reaction [34]. Human Th17 cells express *CCR6*, which is associated with preferential homing to the mucosal sites [28,35,36] in response to its chemokine ligand CCL20 or MIP-3α [37,38]. In murine allergic asthma models, it has been shown that IL-17 exerts its pro-inflammatory effects on the respiratory epithelium in allergic immune reactions by promoting the exacerbation of IL-33-induced neutrophilic inflammation [39]. IL-17 induces the polymeric Ig receptor as a key factor in mucosal immunity [40] and enhances the chemotaxis of B cells during asthma [41].

Allergen-specific immunotherapy (AIT) as the only causal, disease-modifying treatment for allergic diseases with long-term effects [42] aims to restore tolerance to allergens and thus, prevent the disease from spreading to the lower respiratory tract [43]. Recent studies have shown that a subgroup of T cells that expresses both the pro-inflammatory cytokine IL-17 and the regulatory transcription factor FoxP3 are induced during AIT (Zissler, Jakwerth, et al. 2018). These cells are postulated as a transitory subgroup between Th17 cells and regulatory T cells (Tregs), since both populations can trans-differentiate in one, as well as in the other, direction [44,45]. Interestingly, the ratio of anti-inflammatory immune cells, such as regulatory B cells, to pro-inflammatory components, such as the number of Th17 cells, in relation to an earlier time point in long-term AIT, correlates with clinical success in terms of symptom reduction after three years of therapy [28]. Regulatory (*IL10*, *PD-L1*) and pro-inflammatory (*RORC*, *IL17C*, *IL4*, *IL5*, *IL13*) gene expression signatures in the nasal mucosa confirmed the peripheral processes locally in the nasal mucosa of AIT-treated allergic rhinitis patients.

Complex interactions exist between epithelial cells and innate or adaptive immune cells in the airways to maintain host defense. Sophisticated transcriptomic technologies have improved our view of the cellular landscape in the human airways, but inherent limitations and many unanswered questions remain [23,46]. The loss of fragile cell types in the process of tissue dissociation and sequencing can bias the analysis and variation in bioinformatic pipelines, and cell annotation can make comparisons between different studies difficult, even though methods are rapidly evolving to allow for these types of integrative approaches [47,48,49]. Rare epithelial cell types have attracted much interest, but given their low frequency in the lungs, the challenge is to determine their contribution to various disease pathologies with the hope that we can harness and manipulate particular cells to improve lung health throughout the human lifespan [23,50]. Signals from epithelial cells maintain tissue-resident immune cells and modulate their response to antigen, while conversely, immune cells can directly alter the function and phenotype of airway epithelial cells [17].

### 2.4. Macrophages

Macrophages normally constitute the largest population of immune cells in the airway lumen, as reflected in induced sputum, as well as in airway brushes and BAL [21,46]. In addition to Th2 cells, macrophages are key players in type 2 inflammation, but their metabolic and epigenetic programs remain largely unknown [51]. In chronic type 2 inflammatory airway diseases, including the non-steroidal anti-inflammatory drug (NSAID)-exacerbated respiratory disease (N-ERD), macrophages were shown to produce higher levels of pro-inflammatory fatty acid metabolites, such as acylcarnitines and 5-LOX products, as well as increased chemokine and cytokine levels, more readily upon inflammatory challenge [51]. Some whole transcriptome studies of sputum samples from patients with allergic rhinitis and asthma indicated a disease-related shift in predominantly airway immune cell populations from resident airway macrophages to granulocytes, displaying pro-inflammatory phenotypes and gene expression changes suggestive of impaired phagocytosis [17,18].

The intimate, cross-linked interactions between resident respiratory macrophages and epithelial cells ensure the maintenance of a homeostatic state of immune tolerance to harmless stimuli such as allergens, followed by protective reactions to inhaled pathogens with effective tissue repair [52]. Analyses of cell–cell interactions from lung tissue revealed an intimate crosstalk between epithelial cells and macrophages in the airway lumen at homeostasis [46]. A subset of nerve and airway-associated macrophages, which is distinct from alveolar macrophages, was shown to play a regulatory role in influenza infection [53]. A number of studies investigated this poorly understood crosstalk, focusing on the contribution of dysregulated immunoregulatory interactions at this primary environmental interface to human disease.

Bi-directional interactions between resident airway macrophages and epithelial cells ensure the maintenance of a homeostatic state of immune tolerance to harmless stimuli and appropriate protective responses to inhaled pathogens, with effective tissue repair when required [52]. A mouse model utilizing live confocal microscopy showed that CD11c-expressing alveolar macrophages formed direct connections with alveolar epithelial cells by connexin-containing gap junctions to limit the inflammatory response to LPS [54]. The exploration of cell–cell signaling networks using scRNA-seq data from lung tissue across four mammalian species (including humans) showed crosstalk between epithelial cells and macrophages in the alveolar cell niche at homeostasis. There are few studies that investigate epithelial–macrophage crosstalk in the human airways, yet this is a critically important research area to fully understand how dysregulated immunoregulatory interactions at this environmental interface contribute to human disease [55,56]. Further, the epithelial–immune axis in an inflammatory airway disease can be mirrored by BAL in atopic asthma before and after subsegmental bronchoprovocation with an identified allergen [57]. After aeroallergen challenge, epithelium-derived M-CSF (CSF1) was increased in the lower airway lining fluids of atopic asthmatics. Investigations of an epithelial cell-specific *Csf1* deletion in the transgenic mouse model corrected the allergic airway inflammation. In addition, epithelial M-CSF increased the count of a subpopulation of alveolar dendritic cells that expresses the M-CSF receptor CD115 after allergen exposure and promoted migration to regional lymph nodes [57]. GM-CSF (*CSF2*), which has a nonredundant function on instructing the alveolar macrophage fate, was expressed in AT2 cells [58]. These results suggest interactions between epithelial cells and dendritic cells in the airways to improve antigen presentation and to enhance adaptive allergic responses.

### 2.5. Dendritic Cells

Since airway epithelial cells also play an active role in regulating immune responses, spatial proximity to dendritic cells (DCs) leads to a continuous interaction and modulation of functions between these two cell types. During viral infection, DCs secrete type I, including IFN-α, IFN-β and IFN-ω, and type III interferons, covering IFNλ-1, -2, -3, and -4, which inhibit expression of the class I major histocompatibility complex (MHC-I) upregulated on airway epithelial cells to enhance the antiviral response [59]. These DC-derived cytokine also affects the tight junction proteins of the epithelial cell barrier to increase permeability and increase the infiltration of other immune cells at the site of inflammation [60,61]. However, this interaction is bidirectional. In their barrier function, airway epithelial cells express PRRs (Pathogen Recognition Receptors) and receptors for allergens, causing them to react to antigens and allergens. Thus, the first step in that direction can initiate host–pathogen interaction [62]. Furthermore, the local microenvironment can define the nature of the immune response triggered by the host [63]. Similar to viral infections, activation by house dust mites induces the release of various epithelium-derived mediators, including CCL2 and CCL20, which can attract immature DCs or monocytic progenitors into the airways [64]. Moreover, the interaction between airway epithelial cells and DCs also plays an important role in inducing type 2 responses against allergens [61]. The activation of airway epithelial cells by allergens causes secretion of IL-25, IL-33, and thymic stromal lymphopoietin (TSLP). These epithelial alarmins act on DCs, regulating Th2 responses via enhanced OX40-OX40L interactions [65]. IL-25 induces the secretion of CCL-17, which causes the attraction of Th9 cells during allergic inflammation [66,67]. However, airway epithelial cells can also prevent Th2 responses by the promotion of the Th1 polarization capacity of DCs. This is possible as monocytes are differentiating in the presence of activated epithelial cells, which leads to an increased secretion of IL-12, IL-6 and TNF-α [68]. In addition to chemokines, cytokines are also produced by AECs, damaging associated molecules; alarmins that can be released during epithelial injury or death can also affect the DC function. Mediators such as HM-GB1 (High Mobility Group Box 1 Protein), ATP (adenosine triphosphate), and uric acid in the airways of asthmatics, thought to be the products of dying epithelial cells, may activate DCs to increase inflammation [69,70]. In addition to affecting DC functions during infections, AECs also modulate DC function at homeostasis.

### 2.6. Mast Cells

Mast cells (MCs) are known to contribute to both acute and chronic airway inflammation. The anatomical localization of mast cells in the airway smooth muscle bundles, independent of type 2 inflammation, has been described as a cardinal pathophysiological feature of asthma. The extent of mast cell density in the airways smooth muscle directly correlates to the degree of airway hyperresponsiveness [71]. This allows a potential direct connection to be made between the immune cells and airway physiology. Subgroups of severe asthma are defined by increased smooth muscle mast cells and increased tryptase, independent of type 2 inflammatory markers [18,72,73]. Mast cells are capable of responding to a variety of activation signals, such as FcεRI/IgE cross-linking, and G protein-coupled receptor (GPCR) or cytokine receptor signaling to secrete soluble mediators. The type of activation signal, in particular, determines the composition, duration, and extent of mediator secretion [74,75]. Secreted mast cell mediators such as IL13, histamine, tryptase, and neuropeptides contribute directly to bronchoconstriction and airway inflammation [76]. Upon exposure to inhaled allergens, the epithelium-derived mediators CCL17 and CCL22 attract and recruit ILC2, Th2 cells, basophils, and Tregs by CCR4. Eosinophils and Th2 cells are recruited by the epithelial cell-derived eotaxins CCL11 (eotaxin-1), CCL24 (eotaxin-2), and CCL26 (eotaxin-3), which act through CCR3 receptors [26]. In addition, epithelial cells are able to attract basophils, ILC2s, and Th2 cells by releasing PGD2 that binds to the CRTH2 receptor [18]. IL-4 and IL-13 released by MCs massively nudge the ability of airway epithelial cells to produce cytokines [77]. Like airway epithelial cells, MCs can also produce TSLP, which is overexpressed in the asthmatic airways, promoting the release of Th2 cytokines, including IL-4, IL-5, and IL-13 [77]. After localizing to the submucosal mucous glands, MCs release mediators and cytokines, including histamine, PGD2, LTC4, TNFα, chymase, IL-4, IL-6, and IL-13. In particular, these promote mucus hypersecretion by hyperplastic submucosal cells and epithelial goblet cells [77]. MC-derived IL-13 is the predominant cytokine associated with mucus secretion and promotes airway mucus secretion in asthmatics [25,78]. Moreover, IL-13 signaling promotes the transdifferentiation of ciliated cells into goblet cells [79]. Massive mucus production, which occurs in asthma, is due to increased secretion of the gel-forming MUC5AC, which is the most abundant macromolecule of mucus secreted by the airways [80]. Interaction with IL-13/IL-4Ra activates cytokine receptor-associated Janus kinases (JAKs), which support the phosphorylation of STAT6 [25]. After dimerization, phosphorylated STAT6 is translocated into the nucleus, suppressing the expression of FOXA2, a transcriptional repressor of MUC5AC [25,78], which, however, is also regulated in anti-inflammatory signaling pathways such as secretoglobin1A1 [17,78].

## 3. Role of microRNAs in Epithelial Immunity

Diverse biological processes are regulated by small, non-coding nucleic acids, or so-called microRNAs (miRNAs). The altered biosynthesis or regulation of microRNAs contributes to pathological processes involving epithelial cells secreting small extracellular vesicles [81].

MicroRNAs are currently being considered as potential new biomarkers, as they are secreted by cells into the extracellular space in exosomes, and are therefore relatively stable in body fluids, e.g., in the serum [82,83,84]. However, a minimally invasive technique of sampling, such as obtaining nasal secretions or nasal brushings, would always be preferable for determining biomarkers, as these samples can also be easily taken from children [25]. So far, most studies have focused on serum microRNAs, but few have studied their expression in the airway mucosa [85]. In allergic conditions, characterized by a cytokine milieu dominated by type 2 mediators such as IL-4 and IL-13, epithelial cells can express miRNAs, which can also be related to a decline in lung function parameters [81]. Further, a correlation with airway obstruction and predicted targets relevant for type 2 polarization in children, such as *IKZF1* and *BMPR2*, strongly suggest a role for these miRNAs in the development of asthma. In addition, airway remodeling and loss of epithelial integrity in asthma influences airway obstruction and hyperreactivity, which might be regulated by epithelial small extracellular vesicles via specific miRNAs.

Three miRNAs with functional relevance for the allergic immune reaction have been investigated in connection with allergic respiratory diseases in independent studies. A particular miRNA has been studied in allergic asthma, *miR-19a* [83]. An increased expression of *miR-19a* in T cells of the airways promoted the production of type 2 cytokines [83,86], while on the other hand, a reduced miR-19a expression in the smooth muscle cells of the airways led to enhanced airway remodeling [87]. In addition, the *miR-155*, which is also enriched in the skin of patients with atopic dermatitis [88], was investigated in a murine allergic airway inflammation model. *MiR-155*-deficient mice had reduced levels of IL-33 in the airways and lower ILC2 numbers compared to wild-type mice upon allergen exposure [89]. In DCs, a decreased chemotaxis was observed when *miR-155* was depleted, which in turn could be a linked to the recruitment of CCR6^+^ T cells mentioned above, resulting in the improved characteristics of experimental asthma. Particular attention should also be paid to the let-7 family, which was described as one of the first miRNAs and which restricts IL-13 expression in the airways [90]. The first publication on miRNA expression in the sputum of allergic patients treated with allergen-specific immunotherapy suggested a *miR-3935*-dependent mechanism targeting a receptor of prostaglandin E_2_ (PGE_2_), an important lipid mediator in allergic airway inflammation [18]. Further research is required to mechanistically define and integrate the role of miRNAs derived from airway epithelial cells in processes driving allergic airway inflammation.

## 4. Epithelial Cytokines in Allergic Diseases

### The Concept of Epithelial Polarization

The role of epithelial cells has been highlighted in translational studies and animal asthma models, going well beyond the role of a physical barrier and component of the innate immune system. Different inflammatory milieus, such as those created by type 1- or type 2-directed immune responses, can have different effects on the biology of the airway epithelium and lead to a type 1-like (E1) or type 2-like (E2) polarization of the respective epithelial cells [26,91]. The resulting E1 versus E2 priming concept of airway epithelial cells was named after the causative Th1 versus Th2-derived cytokines [26]. An inhibitory role for IFN-**γ** in asthma pathogenesis at the epithelial level now seems conclusive, suggesting type 1 responses antagonizing type 2 pathways [92,93]. The selective transgenic expression of the IFN-**γ** receptor on the airway epithelium could demonstrate a direct involvement of airway epithelial cells. Independent of the activation of Th2 cells, IFN-**γ** can inhibit mucus secretion, as well as the release of chitinases and eosinophilia [94]. In turn, GATA-3 inhibition may contribute to an increase in T-BET and IFN-**γ** expression levels, further leading to a suppressed allergic phenotype [95]. In addition, an increase in DNA methylation of IFN-γ during allergic sensitization plays an important role [96]. The immunological effects of epithelial differentiation are also becoming increasingly important in connection with sensitization, as well as with the recovery processes and remodeling of the airways, new options for intervention and prevention of lung damage. Mechanisms such as the epithelial cytokine production of CCL-26 (eotaxin-3) and the epithelial-derived alarmins TSLP and IL-33 increasingly focus on the interaction between the airway epithelium and immune cells in allergy research. Thus, epithelial cells also play an active role in the immune response to allergens, viruses, and other pollutants, which via orchestration using the Th1 and Th2 key cytokines IFN-γ and IL-4 [26,91], the epithelial polarization to an E1/E2 response analogous to the Th1-Th2 model of Tim Mossman [97] is completed. 

Due to impaired barrier function of the epithelial cells in the respiratory tract, genetically predisposed individuals are prone to virus infections and the absorption of allergens, even at a young age, leading to a local, allergen-specific type 2 immune response upon the initial contact trigger, and thus, to sensitization [98,99]. Allergen exposure and the subsequent activation of pattern recognition receptors induce an innate immune response through the epithelial cells of the respiratory tract. As a result, the epithelial alarmins TSLP, IL-25, and IL-33, as well as the M-CSF and GM-CSF, are released within a short time after allergen contact [100,101]. In animal models, neutralizing one or more of these epithelial cytokines could reduce certain features of asthmatic airway inflammation, such as eosinophilia, bronchial hyperreactivity, and goblet cell hyperplasia [57,102,103,104,105,106,107,108,109,110,111,112,113]. However, factors such as age, gender, and epigenetic mechanisms also influence the epithelial cytokine response and the individual amplitude of the individual cytokines [114,115,116]. This has been shown in studies of stable dust exposure on farms, where, for example, the Amish way of life leads to a lower level of prevalence and predisposition to asthma [117]. The abundance of epithelial cytokines such as IL-33 and GM-CSF was also reduced, which could be related to the upregulation of the negative regulator of the NFkB pathway, TNFAIP3 (A20), which is acting as a deubiquitinase [118]. However, additional stimuli, such as air pollution, exposure to cigarette smoke, and viral infections can increase epithelial mediator expression. The epithelial cytokines can also activate adaptive type 2 cell-associated immune mechanisms [114,119]: the epithelial cytokines TSLP, IL-25, and IL-33 control the terminal differentiation and activation of effector Th2 cells [120], but innate lymphoid cells 2 (ILC2) [121], eosinophils, and basophils [122,123], which are activated via epithelial TGF-β, can also be influenced [109,124]. Further, alternatively activated macrophages (AAMs) are also activated by epithelial alarmins and can acquire a TNF-dependent inflammatory memory in allergic asthma [125].

The epithelial mediators IL-25 and IL-33 stimulate the proliferation of type 2 cells and ILC2s and influence their OX40L expression [126,127,128]. Conventional type 2 DCs are activated by the upregulation of OX40L and the suppression of IL-12 [129]. Studies in animal models have shown that in reporter mice, the production of epithelial E2-associated cytokines is a specific function of specialized epithelial cells. IL-25 is released by tuft cells of the epithelium in response to environmental and microbial stimuli [130,131]. These cells play a central role via an inductive and feedback loop in the activation of ILC2 cells [132], which in turn secrete IL-13 [18] and thus further stimulate the expansion of a rare, highly specialized epithelial cell species, the tuft cells [133]. In a murine allergen exposure model, the formation of tuft cells could be weakened by knock-out of leukotriene C4 (LTC4) synthase, or the receptor for LTE4, on epithelial cells [107]. PNECs are another specialized, multi-functional epithelial cell type that occurs at the bifurcations of the bronchi, but in patients with asthma (just like tuft cells), these are found in increased numbers [134,135]. As a result of an allergen challenge, PNECs can express the calcitonin gene-related peptide (CGRP), which stimulates the production of IL-5 in ILC2s and further drives the type 2 immune cascade [136]. In addition, the PNECs promote goblet cell metaplasia through the secretion of γ-aminobutyric acid (GABA) [137].

## 5. Broader Distribution of Inflammatory Memory in Epithelial Barrier Tissues

The mechanisms and functional outcomes of how epithelial barrier tissues adapt to environmental exposures are central to the overall health of the organism [138,139]. Storing information from previous exposures—such as those from allergens, toxins, and viruses—to inform future responses as memories within discrete cell types may allow a tissue to encode this information via epigenetic modifications [140], fundamentally altering its future response [141]. Recently, several lines of evidence have highlighted contributions from myeloid cells, lymphoid cells, stromal cells, and epithelial cells to the emerging concepts of inflammatory memory and trained immunity [63,141,142]. The properties of memory include alterations in the baseline, sensitivity, speed, or maximum response upon a recall event [142]. However, within barrier tissues that are faced with myriad exposures to environmental agents, determining whether observed changes are adaptations (i.e., require the initiating trigger for persistence) or memories (i.e., persist in their absence) requires careful exploration and interpretation of models [63]. These adaptations and memories have a direct impact on the chronicity of diseases, such as chronic rhinosinusitis (CRS) or asthma, and may also affect how barrier tissues deal with novel acute challenges such as the currently rampant novel virus SARS-CoV-2.

Type 1 and type 2 immune effector cytokines have been shown to uniquely polarize and also antagonize their mutual effects on human bronchial epithelial cells [26,119,143]. ScRNA-seq studies have extended these findings and have identified a shift from type 1 to type 2 cytokine-induced signatures, with increasing disease severity in CRS. Interestingly, when basal epithelial cells were cultured from these patients, the type 2 signatures persisted ex vivo through serial passage, suggesting an allergic inflammatory memory composed of several IL-4-induced cytokines, even in the absence of cytokine stimulation [143]. A recently published preprint using air liquid interphase cultures from controls and COPD patients identified long-term epithelial defects as measured by barrier dysfunction, impaired polarity, and lineage abnormalities that persisted in vitro beyond ten weeks [144]. In conclusion, there are lessons to be learned from these types of study designs in order to distinguish the most persistent signatures from those that fade, representing an exciting opportunity to link human scRNA-seq studies with functional experiments using stem-cell derived models. These data have profound implications for our understanding of allergic diseases, given that efforts to treat these diseases are generally focused on manipulating cells of the immune system.

It will be of critical importance to accurately understand whether changes identified through bulk or single-cell transcriptomic studies reflect acute or long-term adaptations (i.e., the stimulus persists), or epithelial type 2 memory (i.e., the functional change persists, absent stimulus) in order to inform and design experiments to test for molecular and cellular mechanisms. The hypothesized mechanisms will enable deeper investigations into the specificity, quantity, quality, and persistence of inflammatory memory in barrier tissues [142]. The storage of potential memory within cell subsets that exist as transient, resident, or permanently resident members of a tissue highlights the potential for memory persistence within cell subsets, such as epithelial stem cells and stromal cell niches in barrier tissues [145]. Identifying how these tissue-essential cell types interact with tissue-resident memory lymphocytes and myeloid cell subsets will be of critical importance [142].

Additionally, our view of tissues through the lens of single-cell biology is broadening our view of potential stores of inflammatory memory in barrier tissues, while simultaneously providing exciting and unexpected findings in airway cell subsets [23,46]. scRNA-seq has allowed for the elucidation of rare cell subsets, such as the Foxi1+ pulmonary ionocyte which expresses Cftr [50]. It will be of interest to utilize these signatures to explore the functional contributions of airway ionocytes to respiratory biology in future studies. While much has been learned from the full-tissue profiling of barrier tissues, the enrichment for poorly-captured cell types can also provide important lessons. Applying this approach to human nasal mast cells identified a subepithelial population of CD38^high^CD117^high^ mast cells that is markedly expanded during type 2 allergic inflammatory disease. CD38^high^CD117^high^ mast cells had an intermediate phenotype relative to canonical mast cell subsets and were enriched for proliferation, indicating a local mechanism for mast cell expansion [146]. This work expands on hallmark studies of mast cells in allergic airway diseases which showed that intraepithelial mast cells increase in Type 2-high asthma, and that IL-13 stimulates production of Kit ligand from epithelial cells [147]. Furthermore, epithelial-derived IL-33 induces IL-13 in mast cells, and the co-culture of mast cells with epithelial cells from asthmatic individuals leads to a feed-forward loop [148].

While allergic and viral diseases are often studied separately, it will be of high interest to explore the links between viral and allergic immunity at single-cell resolution in human tissues. New methods applied to sampling modalities, such as nasopharyngeal swabs to recover and profile single-cells during the COVID-19 pandemic, raise interesting possibilities for studying virally-exacerbated respiratory diseases by surveying for viral transcripts within single-cell data, in addition to host gene expression [149,150]. Through this investigation, it has been possible to identify the specific epithelial and immune cell subsets infected by viruses other than SARS-CoV-2.

Beyond scRNA-seq, understanding how memory is stored at the single-cell level will be essential. Mechanisms explored to date range from short-term transcriptional changes and metabolic states to potentially longer-lasting epigenetic modifications [140]. Looking beyond the airway epithelial barrier, pioneering work has identified specific mechanisms of how long noncoding RNAs can regulate innate immune training within topologically associated domains of the genome [151]. This work also highlighted the importance of differences in the capacity for human, but not mouse, cells to be trained based on the UMLILO lncRNA. These mechanisms, paired with and understanding of specific transcription factors [152] and the growing understanding that genomic regulation occurs as “all-or-none” events, provides an important framework for understanding inflammatory memory in barrier tissues [153]. This line of thinking has begun to be applied to epithelial cells, where the maximum fraction of cells responsive to a bacterial PAMP changes in a digital, rather than analog, fashion, based on licensing at the TLR2 promoter [139]. Given the constant exposure to a myriad of viral, bacterial, fungal, and other environmental agents, human studies in particular will need to account for sources of variation due to acute exposure rather than chronic long-term adaptation or memory [154].

## 6. Treatment Options for Inflammatory Status of Airway Epithelial Cells

This vicious cycle represents most of the asthmatic pathologies, however it remains unknown if dexamethasone regulates functions of airway progenitor cells, which are responsible for epithelial repair. Airway inflammation has been identified as the hallmark responsible for most of the symptoms of asthmatic patients [67]. Therefore, anti-inflammatory treatment has been the first choice in the clinical control of asthma. Airway inflammation accompanied by mucus hypersecretion can be controlled using glucocorticoids [155]. The underlying mechanisms of glucocorticoids are believed to be mediated by glucocorticoid receptors, which are ubiquitously expressed on various cell types, including airway epithelial cells and airway fibroblasts [156]. Glucocorticoids reduce the expression of the major mucin *MUC5AC* in primary differentiated normal human bronchial epithelial cells. However, it remains unknown whether glucocorticoids possess effects on key functions of conducting airway epithelial cells. Further, epithelial cells can not only perceive and react to changes in the pro- or anti-inflammatory cytokine milieu, but also show an intrinsic capacity for inflammatory memory [23]. This has been observed in chronic allergic inflammatory diseases such as CRS [143], a type 2-driven chronic respiratory disease that is defined by persistent inflammation of the lining of the nose and sinuses. Commonly, CRS can be classified into a phenotype with NP (CRSwNP) and one without NP (CRSsNP) by the presence of nasal polyps (NP) [157]. Due to underlying mechanisms, CRS can be sub-divided into a type of type 2 and a type of non-type 2 inflammatory immune response. It is further associated with epithelial damage and tissue destruction [158], which can promote additional viral infections [159]. These features are also hallmarks of remodeling in asthma, which often coexists with CRS. It is also known that deterioration in CRS control can lead to an increased number of asthma exacerbations [160]. Treatment of both CRSwNP and asthma is based on anti-inflammatory therapies, including the topical administration of glucocorticoids.

As basal cell hyperplasia is also a key feature of tissue remodeling in this condition; recent studies were performed on human tissue samples collected during ethmoid sinus surgery from subjects with CRS and CRSwNP, as well as nasal brushings from the inferior turbinate of healthy subjects, and from those with CRS and polyps [143]. The most striking disease-related changes to the transcriptomic state were observed in basal cell, differentiating, or secretory cell and glandular cell populations. In polyp samples, a significant basal cell expansion at the expense of epithelial cell diversity was described. Basal cells upregulated genes responsive for the type 2 key cytokines IL-4 and IL-13. Upregulation of a core set of basal transcription factors such as *ATF3*, *KLF5*, and *FOSB* [143], maintaining undifferentiated cell states, was described, responding to anti-IL-4 therapy in sorted polyp basal cells. Therefore, intrinsic changes at an epigenetic level could be responsible for the differences in the polyp basal cell state and may be governed by the local type 2 inflammatory milieu.

## 7. Concluding Remarks and Future Directions

Airway epithelial cells express a variety of receptors that facilitate their detection of pathogenic microbes, environmental agents, and tissue damage. These cells coordinate this information to implement fine-tuned responses to initiate immune responses, mitigate damage, repair tissue, and restore homeostasis. The pre-commitment defined by the cytokine micromilieu not only instructs the upper epithelial layers in the short-term, but also trains their stem cells to remember and respond quicker to repeated allergen exposures in a type 2-dominated cytokine micromilieu. As has been described for both innate and adaptive immune cells, epithelial cells can also be trained and retain long-term consequences, resulting in their enhanced responsiveness.

In addition, whether there are conserved mechanisms of inflammatory memory between different tissue compartments must also be investigated. In stem cells, the inflammatory memory comprises a cohort of cholesterol biosynthetic genes that are remarkably similar to those in epidermal stem cells. An important task for the future will be to define the underlying mechanisms of trained immunity involved in the establishment of inflammatory epithelial memory [140]. The degree of use of memory in stem cells to meet the particular needs of each tissue and its repair process must also be part of these investigations.

In conclusion, much knowledge has been gained on the interplay between epithelial and immune cells in recent years; however, future work is required to better understand and characterize the inflammatory memory in stem cells. These memory domains were defined by the accessibility of chromatin related to epigenetic patterns. However, the fundamental question of what further characterizes their chromatin landscape that leads to long-term dissemination of epigenetic information remains largely an enigma.

## Data Availability

Not applicable.
